# Ankyloglossia in Newborns: Clinical Implications and Management—A Narrative Review

**DOI:** 10.3390/children13040466

**Published:** 2026-03-28

**Authors:** Teresa Edith Ynurrigarro-Medina, Gabriela Torre-Delgadillo, Adriana Torre-Delgadillo, Selene Velázquez-Moreno, Marlen Vitales-Noyola

**Affiliations:** 1 Postgraduate Program in Pediatric Dentistry, Faculty of Dentistry, Autonomous University of San Luis Potosi, San Luis Potosí 78290, Mexico; a266965@alumnos.uaslp.mx (T.E.Y.-M.); gabriela.torre@uaslp.mx (G.T.-D.); selene.velazquez@uaslp.mx (S.V.-M.); 2 Specialty in Orthodontics and Dentomaxillofacial Orthopedics, Faculty of Dentistry, Autonomous University of San Luis Potosi, San Luis Potosí 78290, Mexico; adriana.torre@uaslp.mx

**Keywords:** ankyloglossia, lingual frenulum, newborns, tongue, tongue-tie

## Abstract

**Highlights:**

**What are the main findings?**

- Neonatal ankyloglossia shows a weak correlation between anatomical appearance and functional impairment, underscoring the need for function-based assessment using validated tools and direct observation of breastfeeding.
- Frenotomy can provide rapid relief of maternal nipple pain and short-term improvements in latch when significant functional restriction persists, but evidence for long-term benefits remains limited and heterogeneous.

**What are the implications of the main findings?**

- Clinical management should prioritize multidisciplinary, function-oriented evaluation and shared decision-making with families to avoid both under-treatment and unnecessary procedures.
- Standardized diagnostic criteria, outcome measures, and longitudinal research are essential to establish clear indications and optimize evidence-based care for affected infants.

**Abstract:**

**Background**: Ankyloglossia is a congenital anomaly characterized by restricted tongue mobility due to a short, thick, or tight lingual frenulum. **Methods**: This narrative review synthesizes current concepts on etiology, clinical presentation, diagnostic approaches, functional implications, and management for ankyloglossia in newborns. **Results**: Ankyloglossia can compromise breastfeeding dynamics, manifesting as suboptimal latch, maternal nipple pain, and inefficient milk transfer, and may influence orofacial function if unrecognized. Because anatomical appearance alone does not reliably predict function, evaluation should prioritize structured functional assessments over purely morphological descriptors. Management should be individualized and stepwise, beginning with lactation support and positioning strategies, and progressing to frenotomy when clear functional limitation persists. In appropriately selected cases, timely intervention can improve feeding efficiency and caregiver comfort while minimizing disruptions to early bonding and nutrition. Post-procedure follow-up is important to confirm functional gains and address residual feeding mechanics. **Conclusions**: A coordinated, multidisciplinary approach aligns diagnosis and treatment with the infant’s functional needs and family goals, promoting safe, effective, and patient-centered care.

## 1. Introduction

Ankyloglossia is a congenital craniofacial anomaly in which the lingual frenulum is abnormally short, thickened, or inelastic, limiting tongue mobility and potentially altering oral functions that depend on efficient tongue elevation and protrusion [1]. Beyond its anatomical description, the functional relevance of ankyloglossia in the neonatal period has drawn considerable clinical attention because impaired tongue movement may disrupt the biomechanics of breastfeeding, seal formation, suction, and milk transfer during a developmental window that is critical for infant nutrition and growth [2]. In line with the World Health Organization’s (WHO) recommendation of exclusive breastfeeding for the first six months of life [3], early identification of feeding difficulties linked to restricted lingual function is a clinical priority. Ankyloglossia can compromise this goal through maternal nipple pain, ineffective latch, prolonged feeds, and suboptimal weight gain [4,5]. Notably, an estimated 50–80% of cases are asymptomatic and may not require intervention, underscoring the importance of distinguishing anatomical variants from clinically significant functional impairment [6]. This has motivated the use of structured functional assessment tools, rather than inspection alone, to guide decision-making during the newborn period (e.g., latch quality, transfer efficiency, and maternal pain scores) [6,7]. Despite the widespread adoption of frenotomy as a pragmatic solution in select cases, the overall quality of evidence remains variable, and conclusions about efficacy are mixed, with reports ranging from clear short-term relief of nipple pain and improved latch to limited or uncertain long-term benefits for feeding or speech outcomes [4,7]. Heterogeneity in diagnostic criteria, outcome measures, and follow-up intervals likely contributes to these discrepancies and to the observed practice variability across pediatrics, lactation medicine, otolaryngology, and dentistry [6,7].

Accordingly, this narrative review aims to synthesize and critically evaluate the current evidence regarding ankyloglossia in newborns, including its etiology, diagnostic approaches, functional implications, and management strategies. Particular emphasis is placed on the potential impact of ankyloglossia on breastfeeding, including breastfeeding mechanics, maternal comfort, and early infant growth. Given the complex and dyadic nature of breastfeeding, this review also considers the interaction between infant oral function, maternal anatomy, milk supply, positioning, and behavioral factors that collectively influence feeding success. In light of ongoing debates regarding diagnosis and the potential risk of overtreatment, this review seeks to provide a balanced and clinically oriented synthesis of the available evidence to support evidence-based decision-making in neonatal care. The review critically evaluates the evidence supporting conservative measures—including latch optimization, skilled lactation counseling, positional techniques, and supportive therapies—versus surgical management through frenotomy or frenuloplasty, highlighting indications for intervention, expected short-term benefits, potential risks, and the limitations of current knowledge regarding long-term outcomes such as speech development, dentofacial growth, and airway health. In addition, we examine variability in diagnostic approaches, patient selection, procedural techniques, and outcome reporting that contributes to inconsistent findings across studies. Finally, we identify key gaps in the literature that warrant standardized definitions, validated functional assessment tools, core outcome sets, and adequately powered prospective trials with longitudinal follow-up. Addressing these gaps is essential to harmonize clinical decision-making, reduce practice variability, and optimize individualized, evidence-based care pathways for affected mother–infant dyads.

## 2. Materials and Methods

### 2.1. Study Type

This study is a narrative review that synthesizes current evidence on neonatal ankyloglossia.

### 2.2. Search Strategy

A structured literature search was conducted in the PubMed/MEDLINE database using the following search terms: (“ankyloglossia” OR “tongue-tie”) AND (“newborn” OR “neonate”). The search was limited to articles published in English or Spanish within the last decade.

### 2.3. Eligibility Criteria

Studies were included if they focused on newborns and infants ≤ 1 year of age. Eligible study designs comprised randomized controlled trials, observational studies, systematic reviews, meta-analyses, clinical guidelines, case reports, and narrative reviews addressing aspects relevant to neonatal ankyloglossia. Animal studies were excluded.

### 2.4. Study Selection Process

The initial search identified 222 records. Titles and abstracts were screened to determine relevance to the core topics of this review, including oral cavity anatomy, tongue embryology and anatomy, lingual frenulum function, diagnostic approaches and classification of ankyloglossia, its impact on breastfeeding and craniofacial development, and treatment outcomes. Additionally, the reference lists of selected articles were manually screened (snowballing) to identify further relevant publications. Full-text articles were subsequently assessed for eligibility.

### 2.5. Data Extraction and Synthesis

Data extraction was performed using a thematic approach. Key information related to pathophysiology, clinical findings, diagnostic criteria, functional implications, and treatment outcomes was extracted and organized into thematic sections. The synthesis of the evidence is narrative and interpretive, aiming to provide a comprehensive overview and critical discussion of the existing literature rather than a quantitative aggregation of data.

### 2.6. Quality Considerations

Although author judgment is inherent to narrative reviews, priority was given to recent systematic reviews, clinical guidelines, and large prospective or well-designed observational studies. Other relevant publications were included when they provided important clinical or conceptual contributions to the topic.

### 2.7. Study Selection Results

**Figure 1:**
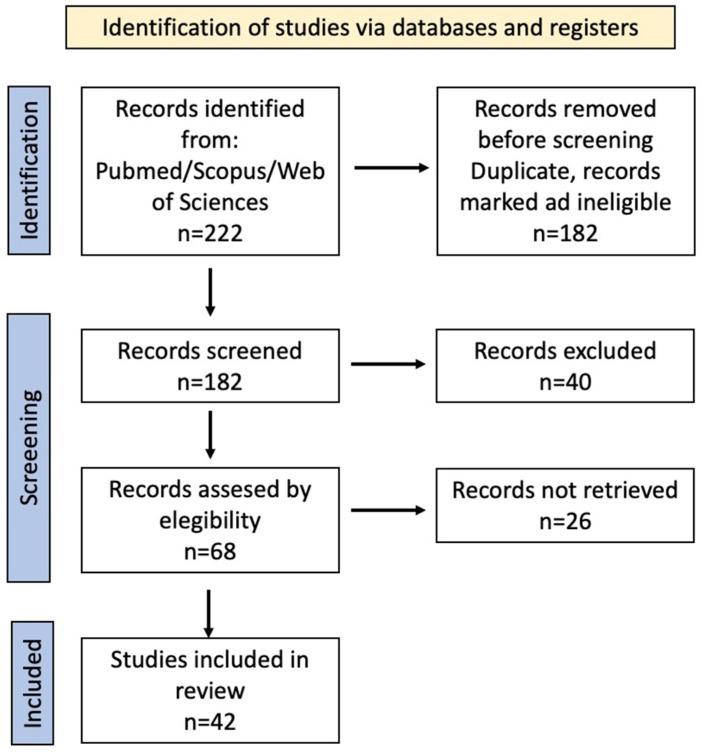
PRISMA diagram for study selection. Arrows indicate the sequential flow of information through the different phases of the review process, including identification, screening, eligibility assessment, and final inclusion of studies.

Following the screening and eligibility process, a total of 42 references were included in the final narrative synthesis. A PRISMA-style flow diagram was constructed to illustrate the study selection process. The initial search identified 222 records in PubMed, and after screening titles and abstracts according to the inclusion and exclusion criteria, 42 articles were considered eligible and included in the final qualitative synthesis (Figure 1).

## 3. Relevant Sections

### 3.1. Etiology

Between gestational weeks 4 and 10, coordinated morphogenesis and programmed cell death sculpt the tongue from the branchial arches. The anterior two-thirds arise from the lateral lingual swellings merging with the tuberculum impar of the first arch, whereas the posterior third derives primarily from the third arch and partially from the fourth arch. This embryologic framework helps contextualize variations in lingual attachment and the spectrum of ankyloglossia phenotypes [8,9]. Importantly, ankyloglossia is not merely a “short frenulum”; it reflects disrupted or incomplete apoptosis of the sublingual connective tissue around week 5, producing a persistently thick, fibrous tether that restricts elevation and protrusion even when tongue length is otherwise normal [10]. Anatomically, the lingual frenulum represents a dynamic midline fold of floor-of-mouth fascia: its visual phenotype varies with tongue posture and traction. Thin, translucent frenula correspond to mobile mucosa gliding over compliant fascia, whereas thick, opaque, blanched frenula indicate mucosa–fascia adherence and are frequently associated with functional limitation on elevation, cupping, and lateralization [11]. Most cases of ankyloglossia appear to occur sporadically [4]; however, a possible genetic component is supported by familial clustering and a male predominance suggestive of X-linked transmission; TBX22 has been implicated as a candidate locus given its established role in craniofacial patterning and its association with X-linked cleft palate, providing a plausible developmental link between orofacial anomalies and restrictive lingual tethering [12,13,14].

### 3.2. Prevalence

Reported prevalence of ankyloglossia in newborns varies widely, typically from 3 to 10%, largely because studies use heterogeneous case definitions (purely anatomical vs. anatomy + function), different screening tools, and diverse examiner training [15,16,17,18]. Estimates tend to be higher when posterior restriction is included and when structured functional instruments are applied, whereas purely visual classifications yield lower rates and greater inter-observer variability [19,20,21]. Most series describe a male predominance and notable variation across settings (well-baby nurseries, lactation clinics, and surgical referrals), suggesting both biologic and ascertainment effects [22]. Prevalence beyond the neonatal period is less consistently reported, in part because many asymptomatic infants are not re-evaluated and because diagnostic thresholds for “clinically significant” restriction shift with age and task demands (feeding and chewing/speech) [23,24,25]. Importantly, apparent increases in tongue-tie diagnoses and frenotomy rates over the last decade likely reflect changes in awareness, referral patterns, and screening practices rather than a true surge in incidence, underscoring the need for standardized, function-anchored criteria when reporting prevalence across populations [26].

### 3.3. Breastfeeding Difficulties

Breastfeeding dysfunction is the most documented issue in symptomatic ankyloglossia [27]. Restricted tongue elevation, lateralization, and cupping impair a deep, effective latch, leading to maternal nipple pain/trauma, poor milk transfer, inadequate infant weight gain, and premature weaning [28,29]. Although the clinical association is strong, causality remains unproven due to the presence of confounding variables and the multifactorial nature of feeding success, as well as the high success of skilled lactation support [7,24]; thus, anatomy alone rarely predicts impairment, and a thorough functional assessment of the breastfeeding dyad is warranted before intervention. A persistently poor latch can alter oral posture and nasal breathing, perpetuating ineffective feeding [30,31]. Newborn feeding relies on the rooting, sucking, and swallowing reflexes; effective milk transfer requires coordinated tongue movements, which are compromised by restriction [4]. Evidence for frenotomy remains inconclusive (while some mothers report less pain and improved feeding, studies are of low quality and inconsistent), though the procedure may help in severe cases [32,33]. Many infants with a short frenulum breastfeed successfully, but they face feeding challenges more often than their peers [32]. Finally, these feeding difficulties are closely linked to breathing patterns because tongue posture influences airway development. All manifestations of ankyloglossia and their clinical relevance are shown in Table 1.

### 3.4. Impact on Breathing

Beyond breastfeeding, a restrictive lingual frenulum can also perturb early orofacial physiology and airway function. In healthy infants, nasal breathing with gently sealed lips and a tongue resting against the transverse palatine folds supports proper palatal molding and maxillary growth, stabilizes mandibular posture, and helps maintain upper-airway patency during sleep [10]. By contrast, infants with a short or inelastic frenulum frequently exhibit a low-tongue posture at rest, slightly parted lips, and a mouth-breathing pattern. This maladaptive rest posture disengages the tongue from the palate, reduces the transverse expansive forces necessary for normal palatal widening, and may promote a deeper, narrower, or high-arched palate, with downstream effects on maxillomandibular development and dental arch form [34,35,36]. Functionally, chronic oral breathing can increase inspiratory flow turbulence, reduce nasal nitric-oxide-mediated antimicrobial and vasoregulatory benefits, and diminish the stabilizing “splinting” effect of the tongue against the hard palate, thereby predisposing to upper-airway collapsibility during quiet sleep [34,35,36]. Over time, these alterations in form and function may contribute to sleep-disordered breathing phenotypes in susceptible children, reinforcing the rationale for early identification of dysfunctional rest posture and targeted conservative interventions (e.g., lactation support to re-establish nasal breathing and palatal tongue rest, as well as myofunctional guidance) before considering surgical release in carefully selected cases [10,34,35,36].

### 3.5. Craniofacial and Dentofacial Development

In typical development, the tongue rests gently against the hard palate, providing lateral and transverse forces that guide maxillary expansion and promote a broad, shallow palatal vault. In infants with ankyloglossia, restriction of tongue elevation disrupts this physiologic tongue–palate contact, favoring a high, narrow (ogival) palate and reduced maxillary transverse width [37,38]. The resulting transverse deficiency can cascade into altered dental arch form, anterior crowding, and posterior crossbite patterns as the deciduous and mixed dentitions emerge. Functionally, a constricted maxilla may reduce nasal cavity volume and increase airflow resistance, encouraging compensatory oral breathing that further perpetuates low-tongue posture and perioral muscular hyperactivity (e.g., mentalis strain), reinforcing the malocclusion cycle. Over time, these interrelated changes in maxillomandibular growth and soft-tissue posture can contribute to Class II tendencies, vertical dysplasia in susceptible phenotypes, and increased upper-airway collapsibility during sleep. Early recognition of dysfunctional rest posture and palatal shape, paired with conservative measures such as lactation optimization, orofacial myofunctional guidance, and vigilant occlusal monitoring, may mitigate downstream dentofacial sequelae, reserving surgical release and, when appropriate, interceptive orthopedic expansion for carefully selected cases [37,38].

### 3.6. Long-Term Considerations: Speech and Occlusion

In older children, the functional profile of ankyloglossia often shifts toward speech and oromotor tasks that require precise, rapid tongue tip elevation and lateralization. Articulatory challenges are most reported for lingual–alveolar and sibilant sounds (/t/, /d/, /l/, /s/, and /r/), which depend on stable palatal contact and fine-grained timing; however, the causal link between tongue-tie and persistent speech sound disorders remains weaker and more heterogeneous than the evidence for breastfeeding difficulties, with many children compensating effectively through alternative articulatory placements [39,40]. Concurrently, altered craniofacial growth patterns associated with low-tongue posture and reduced palatal contact may increase the risk of malocclusions, particularly maxillary transverse deficiency with posterior crossbite, anterior crowding, and, in some phenotypes, anterior open bite, through a combination of skeletal constraint and maladaptive perioral muscle activity [26]. Beyond occlusion and articulation, low-tongue rest posture and habitual oral breathing can intersect with airway physiology, potentially amplifying sleep-disordered breathing risk in predisposed individuals. Taken together, these developmental and functional considerations support a holistic, multidisciplinary approach, integrating lactation support, speech–language pathology, pediatric dentistry/orthodontics, and, when indicated, oral and maxillofacial surgery, centered on functional assessment and goal-directed therapy rather than anatomy alone [26,39,40].

### 3.7. Diagnosis

**Figure 2:**
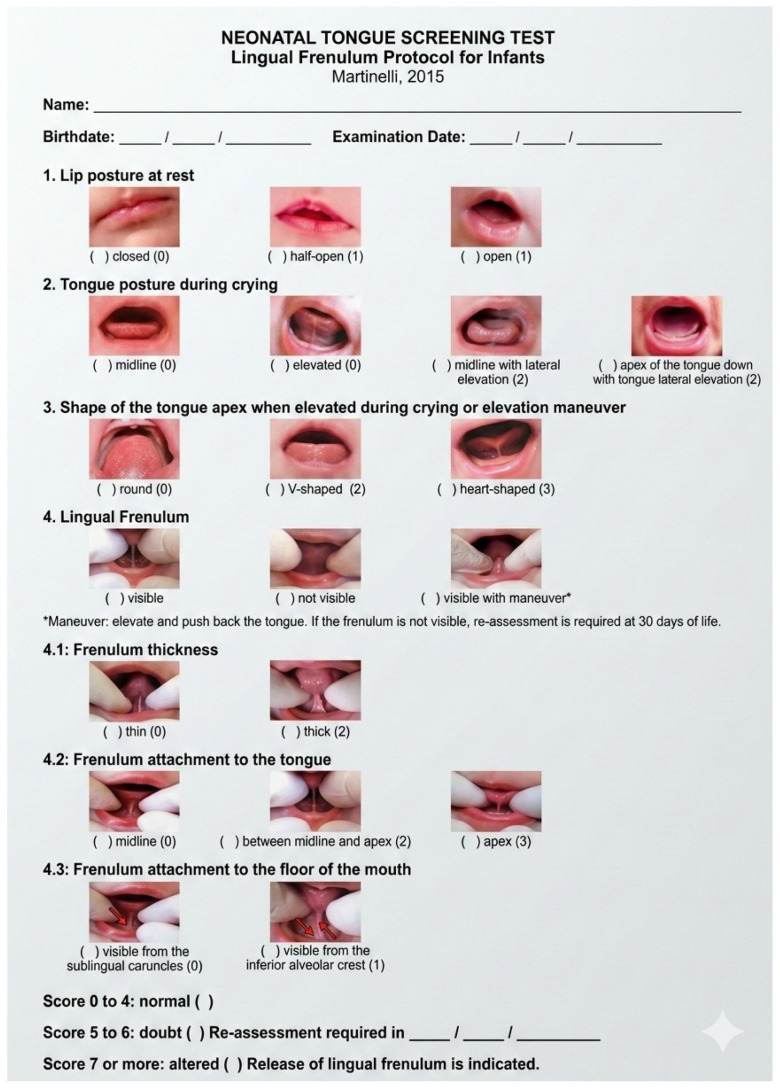
Neonatal tongue screening test using Martinelli’s lingual frenulum protocol for infants. Red arrows indicate the anatomical site of frenulum attachment, highlighting its position relative to the surrounding oral structures.

**Figure 3:**
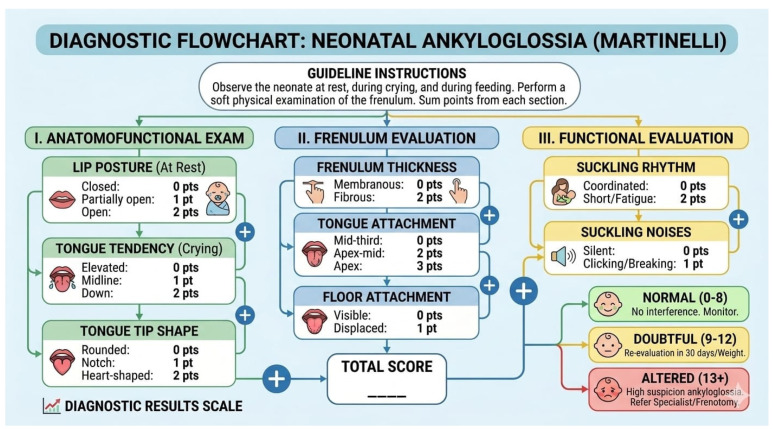
Diagnostic flowchart for the clinical evaluation of neonatal ankyloglossia based on the Martinelli lingual frenulum screening protocol. Arrows indicate the sequence and direction of the diagnostic process across the different evaluation stages. Plus (+) symbols represent the addition of scores from each section to obtain the total score, which determines the final diagnostic classification.

Diagnosis should extend well beyond visual inspection because correlations between frenulum appearance and functional limitation are weak and inconsistent across observers. The Coryllos I–IV classification, anchored in the visible insertion of the frenulum, is widely used in practice, yet it is subjective, lacks validation for predicting breastfeeding or long-term functional outcomes, and does not reliably distinguish infants who will benefit from intervention [41,42]. Accordingly, structured functional instruments are recommended to complement (not replace) anatomical description. The Hazelbaker Assessment Tool for Lingual Frenulum Function (HATLFF) provides a comprehensive appraisal of appearance (anatomy) and mobility (function), can capture posterior restriction, and offers decision thresholds; however, it is more time-consuming and requires training for consistent scoring [43,44,45]. The Bristol Tongue Assessment Tool (BTAT) offers a rapid clinical screen with fewer items and good inter-rater agreement, making it pragmatic for busy neonatal settings, though it is intentionally minimalist and should be paired with direct observation of latch and milk transfer [45,46]. Current best practice is to integrate anatomical grading with standardized functional assessment and observed feeding performance (e.g., latch quality, maternal pain scores, and test-weighing for transfer), thereby aligning diagnosis with clinically meaningful outcomes. The Martinelli Neonatal Tongue Screening test operationalizes this approach within the first 48 h of life, combining anatomy and function into actionable cutoffs (0–4: normal; 5–6: re-evaluate in 30 days; and ≥7: frenotomy indicated), while also furnishing a framework for post-release monitoring to document functional gains and guide adjunctive lactation or myofunctional support (Figure 2 and Table 2) [47]. Embedding these tools into a stepwise clinical pathway (screening, targeted lactation intervention, reassessment, and consideration of release if persistent functional impairment) helps reduce unnecessary procedures and focuses treatment on dyads most likely to benefit (Figure 3) [41,42,43,44,45,46,47].

### 3.8. Treatment

Management of ankyloglossia encompasses stepwise non-surgical and surgical pathways tailored to demonstrable functional need [48,49]. In infants with feeding difficulties, skilled lactation support is the first-line treatment: optimizing latch, positioning, and milk transfer, using asymmetrical latch techniques, brief pre-feed expression, and paced feeding can resolve a substantial proportion of cases without release [48,49,50].

A multidisciplinary assessment, including a lactation consultant, pediatrician, and, when needed, ENT (Ear, Nose, and Throat; otolaryngologist), pediatric dentist, or OMFS (oral and maxillofacial surgery/surgeon), should document objective outcomes (e.g., maternal pain scales, test-weighing, and feeding duration/effectiveness) before any procedural step [48,49,50]. In toddlers and older children, orofacial myofunctional therapy (OMT) is a key adjunct to address low-tongue rest posture, lateralization deficits, and compensatory perioral patterns. When a release is indicated, pre-/postoperative OMT improves motor learning, maintains palatal tongue rest, and reduces scar-related re-tethering compared with surgery alone [51]. Across ages, surgery is reserved for persistent, objectively verified functional impairment, such as refractory painful breastfeeding, consistently poor transfer/weight gain in infants, or documented speech/feeding dysfunction in older children, after skilled conservative care has failed [5,27]. Procedural options include frenotomy (simple division), frenectomy (excision), and frenuloplasty (release with closure and lengthening, e.g., Z-plasty and V-Y plasty), delivered with scissors, electrocautery, or laser platforms [52,53,54,55]. Choice depends on age, phenotype (anterior vs. posterior restriction), tissue thickness, provider expertise, and setting. Laser frenotomy (e.g., CO_2_ and diode) may confer superior hemostasis and shorter operative times, with reports of less intraoperative bleeding and potentially less postoperative discomfort; however, it carries device-specific risks (carbonization and thermal injury), requires appropriate eye protection and smoke evacuation, and generally entails higher cost [56,57]. Regardless of modality, meticulous technique (adequate analgesia, precise depth of release, hemostasis, and early functional re-education) is central to outcomes. When breastfeeding is affected, the strongest evidence supports frenotomy for rapid reduction in maternal nipple pain and short-term improvement in latch/feeding efficiency in appropriately selected dyads [58]. Longer-term effects on growth and speech remain more variable and methodologically heterogeneous. Complications are uncommon and typically minor, such as transient bleeding, localized pain, superficial infection, injury to adjacent structures, or reattachment, with the latter mitigated by adequate release, gentle wound care, and early functional use rather than aggressive stretching [24,49,59]. Clinicians should maintain diagnostic vigilance to avoid anchoring bias: reflux, allergy, hypotonia, craniofacial variability, or neuromotor disorders can mimic or exacerbate feeding challenges and should be considered before attributing symptoms solely to a restrictive frenulum [24,49]. A pragmatic care pathway is: (1) standardized anatomical + functional assessment; (2) targeted lactation/behavioral intervention with objective measures; (3) re-evaluation; and (4) procedure only if clinically meaningful goals remain unmet, followed by structured aftercare (lactation follow-up, with OMT as age-appropriate) to consolidate functional gains [48,49,50,51,52,53,54,55,56,57,58,59].

## 4. Discussion

Diagnosis and management of neonatal ankyloglossia remain debated largely because the relationship between anatomical appearance and functional impairment is inconsistent and infant feeding represents a complex dyadic interaction between the infant and the breastfeeding parent [4,15]. These considerations highlight the importance of adopting a function-oriented clinical framework that integrates anatomical assessment with functional evaluation, including observation of latch, milk transfer, and maternal symptoms. Within this framework, frenotomy may be considered when clinically significant functional impairment persists despite adequate lactation support. Current evidence suggests that the procedure can provide short-term improvements in breastfeeding comfort and latch, particularly through rapid relief of maternal nipple pain. However, variability in diagnostic criteria, patient selection, surgical techniques, and outcome measures continues to limit the strength of conclusions regarding long-term effects on growth, speech, and craniofacial development, underscoring the need for more standardized approaches in both research and clinical practice [16,17,20]. To advance both research quality and clinical care, the field needs: standardized, comprehensive grading systems that combine anatomy and function; core outcome sets that include maternal and infant-centered endpoints (pain, transfer efficiency, weight trajectory, reoperation/reattachment, and need for adjunctive therapy); prospective, adequately powered trials with transparent phenotyping (anterior vs. posterior restriction), clear indications, and technique details (scissors, electrocautery, laser, and frenotomy vs. frenuloplasty); and longitudinal follow-up to evaluate speech, dentofacial growth, airway health, and sleep outcomes [23,24,26]. Clinically, a stepwise pathway—screening, targeted lactation intervention, reassessment, and procedure only if goals remain unmet—minimizes unnecessary releases and focuses treatment on dyads most likely to benefit. Post-procedure, structured aftercare (ongoing lactation support in infants; age-appropriate orofacial myofunctional therapy in older children) helps consolidate functional gains and reduce re-tethering [33].

Finally, shared decision-making with families, grounded in realistic expectations, procedural risks, and available alternatives, should accompany any intervention. Parents and caregivers should receive balanced, evidence-based information regarding the natural variability of breastfeeding difficulties, the potential benefits and limitations of frenotomy, possible complications, and the role of non-surgical approaches such as targeted lactation support. Cultural beliefs, parental anxiety, feeding goals, and the dyadic nature of breastfeeding must also be considered, as these factors strongly influence treatment preferences and satisfaction. Transparent communication helps avoid both under-treatment of infants with genuine functional impairment and overuse of procedures in cases where anatomical findings do not translate into clinically meaningful dysfunction. Harmonizing definitions, measurements, and reporting across studies will enable more reliable prevalence estimates, clearer indications for intervention, and improved comparability of outcomes. The adoption of standardized diagnostic criteria, validated functional assessments, and core outcome sets, including maternal pain, feeding efficiency, infant growth, reattachment rates, and long-term developmental parameters, would substantially strengthen the evidence base. Ultimately, a multidisciplinary, function-oriented approach involving pediatricians, lactation consultants, pediatric dentists, otolaryngologists, speech–language pathologists, and other relevant professionals is essential to ensure individualized care that prioritizes meaningful functional outcomes over anatomical appearance alone and supports optimal health trajectories for both infants and families.

## 5. Conclusions

Neonatal ankyloglossia is a clinically relevant but heterogeneous condition in which anatomical appearance alone does not reliably determine functional impairment or the need for intervention. Given the variability in presentation and the limitations of current diagnostic tools, assessment should prioritize a function-centered approach that integrates clinical evaluation, validated instruments, and lactation support within the breastfeeding dyad. Although frenotomy may provide short-term benefits, particularly in reducing maternal nipple pain and improving breastfeeding, evidence regarding its long-term impact remains inconclusive. Therefore, management should follow a stepwise, multidisciplinary approach, beginning with conservative measures and reserving surgical intervention for cases with persistent functional impairment. Shared decision-making with families is essential to balance potential benefits and risks. Future research should focus on establishing standardized diagnostic criteria and generating high-quality evidence with long-term, clinically meaningful outcomes to support more consistent and evidence-based care.

## Figures and Tables

**Table 1:** Ankyloglossia: manifestations and clinical significance.

Manifestation	Clinical Relevance
Difficulty in sucking	Ineffective latch and poor milk transfer leading to feeding inefficiency
Maternal nipple pain and/or cracked nipples	Secondary to abnormal suction mechanics and poor latch
Prolonged or very frequent feeding sessions	Due to incomplete milk extraction and increased feeding demand
Tongue unable to extend beyond the lower lip	Reflects anterior mobility restriction
Growth delay or poor weight gain	Result of insufficient nutritional intake from ineffective breastfeeding
Recurrent mastitis (mother)	Caused by milk stasis and incomplete breast emptying
Nipple callus (sucking blister)	Hyperkeratosis because of maladaptive suction
Facial asymmetry	Related to abnormal muscle recruitment during feeding
Crooked mouth opening	Compensatory movement to accommodate restricted tongue
Tongue remains low during crying	Failure of tongue elevation and lack of palatal contact
High-arched palate (ogival palate)	Altered craniofacial development due to absent tongue–palate stimulation
Cheek hypertrophy	Compensatory overuse of buccinator muscles during sucking

**Table 2:** Score interpretation of Martinelli’s neonatal tongue screening test.

Total Score	Interpretation	Recommendation
0–4	Normal	No action required
5–6	Borderline/Doubt	Reassessment in 30 days
≥7	Altered	Frenotomy indicated

## Data Availability

No new data were created or analyzed in this study. Data sharing is not applicable to this article.

## Abbreviations

The following abbreviations are used in this manuscript:

WHO	World Health Organization
HATLFF	Hazelbaker Assessment Tool for Lingual Frenulum Function
BTAT	The Bristol Tongue Assessment Tool
OMT	Orofacial Myofunctional Therapy
ENT	Ear, Nose, and Throat; Otolaryngologist
OMFS	Oral and Maxillofacial Surgery/Surgeon
